# Extended-spectrum beta-lactamase, AmpC, and carbapenemase-producing Gram-negative wastewater isolates from Aotearoa New Zealand

**DOI:** 10.1128/mra.00131-24

**Published:** 2024-04-02

**Authors:** Christina Straub, Louise Weaver, Angela J. Cornelius, Erin McGill, Kristin Dyet, Isabelle Pattis

**Affiliations:** 1 Institute of Environmental Science and Research Ltd., Health & Environment, Auckland, New Zealand; 2 University of Vienna, Centre for Microbiology and Environmental Systems Science, Vienna, Austria; 3 Genomics Aotearoa, Dunedin, New Zealand; 4 Institute of Environmental Science and Research Ltd., Health & Environment, Christchurch, New Zealand; 5 Institute of Environmental Science and Research Ltd., Health & Environment, Wellington, New Zealand; DOE Joint Genome Institute, Berkeley, California, USA

**Keywords:** AMR, wastewater

## Abstract

Extended-spectrum beta-lactamase, AmpC, and carbapenemase-producing bacteria were isolated from raw sewage, effluent, oxidation pond water, and sediment from a wastewater treatment plant in Aotearoa New Zealand. Here, we report the assemblies of 17 isolates belonging to the species *Aeromonas veronii*, *Aeromonas hydrophila*, *Enterobacter cloacae*, *Enterobacter soli*, *Lelliottia amnigena*, *Aeromonas caviae*, *Escherichia coli*, *Pseudomonas moraviensis*, *Pseudomonas putida*, and *Kluyvera ascorbata*.

## ANNOUNCEMENT

Environmental and wastewater monitoring plays a crucial role in stemming the global spread of antimicrobial resistance (AMR) (1). Wastewater treatment plants (WWTP) can create favorable conditions for the transfer of AMR genes between bacteria (2), while they are also an important barrier in preventing the spread of AMR into the environment (3). Here, we isolated antibiotic-resistant bacteria from raw sewage, effluent, oxidation pond water, and sediment from a municipal WWTP in Christchurch, New Zealand, in November (raw sewage and effluent) and December 2019 (pond water and sediment). Sampling times were staggered to account for the time of travel through the WWTP.

Carbapenem-resistant isolates (CARB) were isolated using CHROMagar mSuperCARBA (CA-CARBA, Fort Richard Laboratories, NZ); extended-spectrum beta-lactamase (ESBL) and AmpC producers were isolated using CHROMagar ESBL (CA-ESBL, Fort Richard Laboratories, NZ). Plates were incubated at 37°C for 21 ± 2 h.

Colonies of interest were re-streaked twice onto the respective chromogenic agars. Isolates were then re-streaked on tryptic soy agar and stored in tryptic soy broth and 20% glycerol at −80°C. Genomic DNA was extracted from a single colony using the High Pure PCR Template Preparation Kit (Roche Molecular Biochemicals, IN, USA) following the manufacturer’s instructions.

Thirteen isolates were assembled using a hybrid approach utilizing long-read sequencing (LR) for assembly and short-read sequencing (SR) for polishing (Table 1). The remaining four isolates were sequenced using solely LR. For SR, isolates were sequenced on a Illumina NextSeq platform (2 × 150 bp paired-end reads) using Illumina libraries (Nextera-XT DNA library preparation) and protocols. LR libraries were prepared using the Rapid Barcoding Kit (SQK-LSK109, Oxford Nanopore Technologies, Oxford, England). Sequencing was performed as a single run (CARB25), and two multiplex runs on a MinION, using MinKNOW v.20.10.3 and MinKNOW Core v.4.1.2 software, on a FLO-FLG001 flow cell (CARB25), a FLO-MIN106 flow cell (multiplex run 1), and a FLO-MIN112 flow cell (multiplex run 2). Base calling was performed using Guppy 4.2.2+effbaf8 (CARB25), Guppy v.5.0.11+2b6dbff (multiplex run 1), and Guppy 6.1.5+446c35524 (multiplex run 2) with the super high accuracy model. Adapter and quality trimming of reads was performed with Trimmomatic v.0.39 (4) (SR) or Filtlong (https://github.com/rrwick/Filtlong, minimum length 5,000 bp, minimum mean quality 85). This was followed by assembly with Flye v.2.8-1 (5). Reads were then mapped to the Flye assembly using minimap2 v.2.17 (6), and polished with Racon v.1.4.2 (https://github.com/isovic/racon) and medaka v.1.4.3. (https://github.com/nanoporetech/medaka). In addition, short-read assemblies were polished using Unicycler polish v.0.4.8 (7). Circularization of genomes and coverage was determined based on the output of Flye. Quast v.5.0.2 (8) was used to determine genome statistics. Default parameters were used for all software, unless otherwise specified. Abricate (https://github.com/tseemann/abricate) was used to identify plasmids using the PlasmidFinder database (9) (updated 1 November 2022). Taxonomic assignment was based on 16S rRNA gene sequences and using average nucleotide identity (10).

**TABLE 1:** Genome statistics for all 17 isolates^*a*^

Isolate	Source	Species	Contigs	Genome length (bp)	Largest contig (bp)	N50	GC (%)	Coverage	N plasmids	Assembly accession	SRA accession(s)	N reads (SR)	Avg length (SR)	Reads (LR)	Avg length (LR)
CARB02	RS	*Aeromonas veronii*	6	5,245,896	5,093,840	5,093,840	58.24	102	1	JAZKKY000000000	SRR22721384, SRR22721379	7,380,494	142	4,000	4,558
CARB12	E	*Aeromonas hydrophila*	3	4,895,929	4,882,314	4,882,314	61.2	67	0	JAZKKZ000000000	SRR22721383, SRR22721378	7,074,518	149	4,000	8,844
CARB25	E	*Aeromonas veronii*	1	4,808,255	4,808,255	4,808,255	58.45	66	0	CP145798	SRR22721372, SRR22721377	6,413,400	148	4,000	10,688
CARB33*	RS	*Escherichia coli*	3	5331114	5,164,516	5,164,516	50.67	137	2	JAZKKU000000000	SRR26562385	NA	NA	135,655	18,067
CARB46	PW	*Enterobacter* sp. HK169	2	4,810,483	4,637,538	4,637,538	55.92	216	0	JAZKLA000000000	SRR22721365, SRR22721376	4,784,294	149	1,000	5,019
CARB53	PW	*Lelliottia amnigena*	2	4,675,495	4,530,957	4,530,957	52.68	152	1	JAZKLB000000000	SRR22721364, SRR22721375	5,582,300	149	4,000	10,365
CARB60	S	*Enterobacter cloacae* complex	2	5,030,268	4,850,842	4,850,842	55.64	114	1	JAZKLC000000000	SRR22721363, SRR22721374	5,429,894	148	4,000	10,464
ESBL03	RS	*Enterobacter soli*	4	5,311,732	4,912,840	4,912,840	53.35	190	2	JAZKLD000000000	SRR22721362, SRR22721373	4,214,400	148	4,000	10,304
ESBL07	E	*Aeromonas caviae*	3	5,188,023	5,105,001	5,105,001	60.4	118	0	JAZKLE000000000	SRR22721361, SRR22721371	6,980,596	147	4,000	6,550
ESBL09*	E	*Kluyvera ascorbata*	6	5641422	5,042,089	5,042,089	53.56	149	3	JAZKKV000000000	SRR26562384	NA	NA	199,299	17,946
ESBL12*	RS	*Kluyvera ascorbata*	5	5641433	5,043,354	5,043,354	53.56	160	3	JAZKKW000000000	SRR26562383	NA	NA	197,013	18,073
ESBL13	RS	*Escherichia coli*	2	4,845,263	4,791,602	4,791,602	50.79	110	1	JAZKLF000000000	SRR22721360, SRR22721370	6,886,760	147	4,000	9,450
ESBL19*	E	*Escherichia coli*	6	5044947	4,803,132	4,803,132	50.93	123	3	JAZKKX000000000	SRR26562382	NA	NA	114,642	17,295
ESBL22	E	*Escherichia coli*	3	5,097,165	5,052,681	5,052,681	50.66	106	2	JAZKLG000000000	SRR22721359, SRR22721369	10,293,180	141	4,000	8,891
ESBL32	PW	*Pseudomonas moraviensis*	2	5,919,079	5,873,336	5,873,336	60.05	90	0	JAZKLH000000000	SRR22721382, SRR22721368	8,980,338	146	4,000	10,519
ESBL35	PW	*Lelliottia amnigena*	2	4,675,096	4,530,550	4,530,550	52.68	98	1	JAZKLI000000000	SRR22721381, SRR22721367	7,632,878	147	4,000	10,829
ESBL64	PW	*Pseudomonas putida* group	1	5,873,281	5,873,281	5,873,281	61.98	52	0	CP145799	SRR22721380, SRR22721366	7,453,584	148	4,000	10,299

^*a*^ The use of the specific isolation agar is reflected in the isolate names. Asterisk indicates long-read sequencing only. Source, sample types (RS, raw sewage; E, effluent; PW, pond water; S, sediment); SR, short reads; LR, long reads. SRA, sequence read archive. NA, not available. Where both SR and LR data are available, both SRA accessions are listed.

Circularized genomes were obtained for all 17 sequenced isolates, with the number of contigs ranging from one to six (Table 1). Of these isolates, six did not carry any plasmids, five carried one plasmid, and the remaining six carried multiple plasmids. Genome depth of coverage ranged from 52× to 216×. The complete genomes produced in this study will provide a valuable resource for those species that are underrepresented on the publicly available genome database from National Center for Biotechnology Information (NCBI), such as *Lelliottia amnigena, Pseudomonas moraviensis, Enterobacter soli,* and *Aeromonas caviae*.

## Data Availability

All raw reads and genome assemblies have been submitted to NCBI under Bioproject #PRJNA904380. Assembly and SRA accession numbers are provided in (Table 1).
